# Relationship between total phenolic contents and biological properties of propolis from 20 different regions in South Korea

**DOI:** 10.1186/s12906-016-1043-y

**Published:** 2016-02-18

**Authors:** Xue Wang, Karuppasamy Sankarapandian, Yizhe Cheng, Soon Ok Woo, Hyung Wook Kwon, Haribalan Perumalsamy, Young-Joon Ahn

**Affiliations:** Entomology Major, Department of Agriculture Biotechnology, Seoul National University, Seoul, 151-921 South Korea; Research Institute of Agriculture and Life Sciences, Seoul National University, Seoul, 151-921 South Korea; WCU Biomodulation Major, Department of Agricultural Biotechnology, Seoul National University, Seoul, 151-921 South Korea; National Academy of Agricultural Science, Rural Development Administration, Jeonju, 560-500 South Korea

**Keywords:** Korean propolis, BACE-1, Antioxidant, Acetylcholinesterase inhibitory activity, Antiproliferative activity, Antibacterial activity, Anti-human rhinovirus activity, Polyphenol, Flavonoid, Correlation

## Abstract

**Background:**

Propolis (or bee glue), collected from botanical sources by honey bee, has been used as a popular natural remedies in folk medicine throughout the world. This study was conducted to assess growth inhibitory effects of ethanol extracts of propolis (EEPs) from 20 different regions in South Korea on human intestinal bacteria as well as their human β-amyloid precursor cleavage enzyme (BACE-1), acetylcholinesterase (AChE) inhibitory, antioxidant, antiproliferative, and anti-human rhinovirus activities.

**Methods:**

The Bonferroni multiple-comparison method was used to test for significant differences in total polyphenol and flavonoid contents among EEP samples using SAS 9.13 program. Correlation coefficient (*r*) analysis of the biological activities of EEP samples was determined using their 50 % inhibition concentration or minimal inhibitory concentration values and their polyphenol or flavonoid contents in 20 native Korean EEP samples.

**Results:**

The amounts of total polyphenol and flavonoids in the Korean EEP samples ranged from 49 to 239 mg gallic acid equivalent (GAE)/g EEP (Brazilian, Chinese, and Australian samples, 127–142 mg GAE/g EEP) and from 21 to 50 mg quercetin equivalent (QE)/g EEP (Brazilian, Chinese, and Australian samples, 33–53 mg QE/g EEP), respectively. Correlation coefficient analysis showed that total polyphenol contents may be negatively correlated with 2,2-diphenyl-1-picrylhydrazyl free radical scavenging activity (*r* = −0.872) and total flavonoid content has no correlation with the activity (*r* = 0.071). No direct correlation between BACE-1 inhibition, AChE inhibition, or antiproliferative activity and total polyphenol or total flavonoid content in Korean EEP samples was found. Gram-positive and Gram-negative bacteria were observed to have different degrees of antimicrobial susceptibility to the EEP samples examined, although ciprofloxacin susceptibility among the bacterial groups did not differ greatly.

**Conclusions:**

Further studies will warrant possible applications of propolis as potential therapeutic BACE-1 blocker, antioxidant, antiproliferative agent, and antimicrobial agent.

## Background

Propolis (or bee glue), a strongly adhesive, resinous natural substance collected from botanical sources (branches, flowers, pollen, and buds) by honey bee (*Apis mellifera* L.) [1], has been used as a popular natural remedy in folk medicine throughout the world. Bees use propolis to seal holes in their honeycombs, smooth out the internal walls, and protect the entrance toward intruders [2, 3]. Propolis is generally composed of 50 % resin and balsam, 30 % wax, 10 % essential and aromatic oils, 5 % pollen, and 5 % various other substances [3], although the precise composition of raw propolis varies with several factors such as botanical source and geographical zones [1–3].

Propolis has been reported to possess a broad spectrum of biological activities, such as anticancer, anticomplement, antihypertensive, antihyperalgesic, hepatoprotective, anti-inflammatory, antioxidant, immunomodulatory, antimicrobial, and antiparasite properties [1, 3–5]. The chemical variability, aroma, and color (brown, green, red, and yellow) of propolis significantly depend on its botanical source, age of the honey preparation, geographical zones, and collection season [2, 6, 7]. In Brazil, propolis from the southeast region and Amazon contains chiefly phenylated phenylpropanoids and polyprenylated benzophenones, respectively, whereas geranyl flavonones have been reported for propolis from the Pacific region, such as Taiwan and Okinawa [7]. Propolis from eastern Mediterranean regions, such as Greece, Crete, and Turkey, may contain predominantly diterpenoids [7]. The variation in the chemical composition of propolis from different origin also causes the diverse biological activities [7]. More than 300 constituents, including aromatic acids and esters, flavonoids (chalcones, flavanones, flavones, flavonols, and dihydroflavonols), waxy acids and terpenoids, were isolated from raw propolis [1, 2]. In Brazil, 12 distinct groups of propolis have been classified according to their botanical origin and biological properties [8]. Very little work exists in relation to biological properties of native Korean propolis, although the antioxidant activity of propolis from several regions in South Korea have been described by Ahn et al. [9].

The aim of the current study was to assess total polyphenol and flavonoid contents as well as the 2,2-diphenyl-1-picrylhydrazyl (DPPH) free radical scavenging, antiproliferative, anti-human rhinovirus (HRV), human β-amyloid precursor cleavage enzyme (BACE-1), and human acetylcholinesterase (AChE) inhibitory activities of ethanol extracts of propolis (EEPs) from 20 different regions in South Korea. In addition, the growth inhibitory effects of the EEP samples on five harmful intestinal bacteria, two nonpathogenic intestinal bacteria, six lactic acid-producing bacteria, and an acidulating bacterium, including Gram-positive bacteria and Gram-negative bacteria, using a microtiter plate-based bioassay and compared with those of ciprofloxacin, a second-generation fluoroquinolone antibiotic with a broad spectrum [10]. The biological activities of the Korean EEP samples were compared with those of EEP samples from Brazil with diverse chemical composition, Australia with various biologically active flavonoids, phenylpropanoids, and stilbenes and prenylated stilbenes, and China with chemical profiles similar to Korean propolis [11]. A correlation between total polyphenol or flavonoid content and biological activities is also discussed.

## Methods

### Materials

Ascorbic acid, (3-(4,5-dimethylthiazol-2-yl)-2,5-diphenyl tetrazolium bromide) (MTT), DPPH, gallic acid, quercetin, and sulforhodamine B (SRB) were purchased from Sigma-Aldrich (St. Louis, MO). 5,5-Dithiobis(2-nitrobenzoic acid) (DTNB), Folin-Ciocalteu’s phenol reagent, and acetylthiocholine iodide (ATChI) were purchased from Sigma-Aldrich. Commercially available antibiotic ciprofloxacin, anticancer agent cisplatin, and AChE inhibitors donepezil hydrochloride, huperzine A and tacrine were supplied by Sigma-Aldrich. Ribavirin was purchased from Tokyo Chemical Industry (Tokyo, Japan). Anitbiotic-antimycotic was purchased from Invitrogen (Grand Island, NY). Recombinant human BACE-1 and fluorogenic peptide substrate (FPS) Mca-SEVNLDAEFRK (Dnp) RR-NH_2_ were purchased from R&D system (Minneapolis, MN). Brain Heart Infusion (BHI) broth and Eggerth-Gagnon (EG) agar were purchased from Becton, Dickinson and Company (Sparks, MD) and Eiken Chemical (Tokyo, Japan), respectively. Minimum essential medium (MEM), RPMI 1640 medium, and fetal bovine serum (FBS) were supplied by Life Technologies (Grand Island, NY). All of the other chemicals and reagents used in this study were of reagent-grade quality and available commercially.

### Propolis samples and extraction

The 20 native Korean propolis samples (P1–20) used in this study are listed in Table 1, along with coordinates. The samples were collected as the crude materials by beekeepers in various regions of South Korea. Propolis samples from Australia, Brazil, and China were purchased from Aussia Pharma (Silverwater, Australia), Uniflora Apicultores Associados (Olimpia, Brazil), and KangSiNong Biotechnology (Wuhan, China), respectively. Because EEP is one of the richest sources of phenolic acids and flavonoids [12], these propolis samples were extracted with ethanol at room temperature for 1 day, and filtered. The combined filtrated was concentrated to dryness by rotary evaporation at 40 °C. The ethanol extracts were kept at −20 °C until use.

**Table 1:** Propolis samples supplied by 20 different apiaries in various geographic regions of South Korea

Sample no.	Apiary site (Province)	Coordinates
P1	Anseong (Gyeonggi)	37°00'30"N, 127°16'30"E
P2	Icheon (Gyeonggi)	37°15'50"N, 127°29'03"E
P3	Yangpyeong (Gyeonggi)	37°29'32"N, 127°29'16"E
P4	Goyang (Gyeonggi)	37°39'30"N, 126°49'50"E
P5	Wonju (Gangwon)	37°20'15"N, 127°56'47"E
P6	Goesan (Chungbuk)	36°48'45"N, 127°47'20"E
P7	Chungju (Chungbuk)	36°58'12"N, 127°57'09"E
P8	Daejeon	36°22'08"N, 127°22'27"E
P9	Dangjin (Chungnam)	36°53'54"N, 126°37'51"E
P10	Goryeong (Gyeongbuk)	35°43'36"N, 128°15'56"E
P11	Uljin (Gyeongbuk)	36°59'30"N, 129°24'46"E
P12	Jinju (Gyeongnam)	35°09'49"N, 128°02'24"E
P13	Changnyeong (Gyeongnam)	35°32'52"N, 128°29'35"E
P14	Geochang (Gyeongnam)	35°41'19"N, 127°54'44"E
P15	Imsil (Jeonbuk)	35°36'44"N, 127°17'07"E
P16	Buan (Jeonbuk)	35°43'46"N, 126°42'59"E
P17	Jeonju (Jeonbuk)	35°49'17"N, 127°09'17"E
P18	Gunsan (Jeonbuk)	35°58'06"N, 126°44'14"E
P19	Gwangju	35°09'35"N, 126°51'11"E
P20	Jeju (Jeju)	33°14'46"N, 126°33'55"E

### Determination of total polyphenol and flavonoid contents

Total polyphenol contents in EEP samples were determined using Folin–Ciocalteu colorimetric method described by Zongo et al. [13] with slight modifications. In brief, 100 μL of 0.2 N Folin–Ciocalteu reagent was added to 96-well plate (SPL Life Sciences, Pocheon, Gyeonggi, South Korea) containing 20 μL of each EEP in 80 % ethanol for 5 min at room temperature in darkness. The 80 μL of sodium carbonate (75 g/L) was added to each well, and the plate was then incubated for 30 min at room temperature with slightly shaking in darkness. The absorbance was determined at 735 nm using a VersaMax microplate reader with SoftMax Pro 5 Software (serial no. SMP500-18672-LWHU) (Molecular Devices, Sunnyvale, CA). EEPs were evaluated at the final concentration of 100 μg/mL. Gallic acid (0–62.5 μg/mL) was applied as the standard, and the calibration equation was Y = 0.0454 X – 0.0056 (R^2^ = 0.9993), where X is the gallic acid concentration in μg/mL and Y is the absorbance at 735 nm. Total polyphenol contents were expressed as mg of gallic acid equivalents (GAE) per g of EEP samples.

Total flavonoid contents in EEP samples were determined using AlCl_3_ colorimetric method [13] adapted to 96-well plate. In brief, 100 μL of 2 % AlCl_3_ was added to 100 μL of each EEP in 75 % ethanol, and the plate was then incubated for 15 min at room temperature in darkness. The absorbance was determined at 435 nm using a microplate reader stated previously. EEPs were evaluated at the final concentration of 100 μg/mL. Quercetin (0–50 μg/mL) was used as the standard, and the calibration equation was Y = 0.0343 X + 0.0177 (R^2^ = 0.9995), where X is the quercetin concentration in μg/mL, and Y is the absorbance measured at 435 nm. Total flavonoid contents were expressed as mg of quercetin equivalents (QE) per g of EEP samples.

### Fluorescence resonance energy transfer enzyme assay

The BACE-1 inhibitory activity of all EEP samples was evaluated according to the methods of Lv et al. [14] and Wang et al. [15]. The assay mixtures consisted of 1 μL of 0.5 μg/μL recombinant human BACE-1, 0.75 μL of 2.5 μg/μL FPS, 47.25 μL of 50 mM sodium acetate (pH 4.5), and EEP (10–2000 μg/mL) in 2 % dimethyl sulfoxide (DMSO). They were incubated for 1 h at 25 °C in darkness, followed by adding 16.6 μL of 2.5 M sodium acetate to terminate the reaction. The intensity of fluorescence was determined using a SpectraMax Gemini XS plate reader with Softmax Pro PC/MAC Software (serial no. US 02947) (Molecular Devices, Sunnyvale, CA) at 355 nm excitation and 405 nm emission at room temperature. The inhibition percentage was calculated according to the formula: % inhibition = 100 – [(*F*_*S*_ – *F*_*S0*_)/(*F*_*C*_ – *F*_*C0*_)] × 100, where *F*_*S*_ and *F*_*S0*_ are the fluorescence of samples at 60 min and zero time, and *F*_*C*_ and *F*_*C0*_ are the fluorescence of control at 60 min and zero time, respectively [13].

### Acetylcholinesterase inhibition assay

The assay procedure was performed using the recombinant human AChE (R&D system, Minneapolis, MN) according to the manufacture’s protocol. In brief, the reaction mixture consisted of 50 μL of AChE (0.044 μg/mL in 0.1 M sodium phosphate buffer (pH 7.5)) and EEP (10–2000 μg/mL) in 1 % DMSO. The reaction was started by adding 50 μL substrate/DNTB mixture (800 μM ATChI in assay buffer containing 400 μM DTNB). The reaction mixture was incubated at 37 °C for 1 h. The absorbance was recorded at 410 nm using a VersaMax microplate reader. The AChE inhibitors donepezil hydrochloride, tacrine, and huperzine A [16] served as positive controls and were likewise formulated.

### DPPH radical scavenging assay

DPPH free radical scavenging activity of all EEP samples was evaluated according to the method described by Blois [17] with minor modifications. In brief, 100 μL of 0.4 mM DPPH methanl solution was added to 96-well plate containing each EEP sample in methanol. Based on the preliminary test results, the radical scavenging activity of each EEP sample was determined with five to seven concentrations ranging from 5 to 2000 μg/mL. After incubation for 30 min at room temperature in darkness, the absorbance was measured at 518 nm by using a VersaMax microplate reader. Ascorbic acid served as a positive control and was likewise formulated. The radical scavenging ability was calculated according to the formula: % DPPH free radical scavenging activity = (1 – *A*_*s*_/*A*_*c*_) × 100, where *A*_*c*_ is the absorbance of the control (without sample) and *A*_*s*_ is the absorbance of the sample.

### Cancer cell lines and cell proliferation assay

Three human cancer cell lines used in this study were as follows: PC-3 (human prostate adenocarcinoma cell line) and MCF-7 (human breast adenocarcinoma cell line) purchased from the Korean Cell Line Bank (Seoul, South Korea); A549 (human lung carcinoma cell line) purchased from the American Type Culture Collection (ATCC) (Manassas, VA). The PC-3 and MCF-7 cell lines were cultured with RPMI 1640 containing 10 % FBS and 1 % antibiotic-antimycotic solution under 5 % CO_2_ and 95 % air at 37 °C, whereas A549 cell line was cultured with MEM containing 10 % FBS, 1 % antibiotic-antimycotic solution, and 1 % glutamine. Cells were grown in SPL Life Science cell culture dishes.

The antiproliferative activity of all EEP samples toward the human cancer cell lines examined was evaluated using a MTT assay described by Morgan [18]. In brief, MTT was dissolved in phosphate-buffered saline (PBS) (pH 7.4) at 5 mg/mL and sterile-filtered. Cells were plated at 2 × 10^4^ cells per well in 100 μL of complete culture medium containing the test EEP samples (dissolved in DMSO Hybri-Max) in 96-well culture plates. The final concentration of DMSO Hybri-Max in all assays was 0.1 % or less. Based on the preliminary test results, the antiproliferative activity of each EEP sample was determined with five to six concentrations ranging from 15 to 1000 μg/mL. The culture plates were incubated for 2 days in a 37 °C in a humidified atmosphere of 5 % CO_2_. The plates were then washed with 100 μL PBS. The 100 μL medium containing 0.05 % MTT was added to each well and then incubated for 4 h at the same condition stated previously. MTT solution was removed and 200 μL DMSO was added to each well. Finally, the plate was shaken for 10 min to dissolve the purple formazan crystals formed. Cisplatin served as positive controls and was similarly formulated. Negative controls only consisted of the DMSO solution only. The optical density values were recorded using a VersaMax microplate reader at a 560 nm and a 670 nm reference.

### Human rhinovirus serotypes and antiviral assay

A human epithelial adenocarcinoma cervix cell line HeLa (ATCC CCL-2) was maintained in MEM supplemented with 10 % FBS and 0.01 % antibiotic-antimycotic solution in a humidified incubator (37 °C and 5 % CO_2_) [19, 20]. HRV-2 (ATCC VR-1112AS/GP) and HRV-4 (ATCC VR-1114AS/GP) were propagated in HeLa cells at 37 °C. Virus titers were determined by using cytopathic effects (CPE) in HeLa cells and were expressed as 50 % cell culture infective dose (CCID_50_) per mL, as described previously [19, 20].

The anti-HRV activity of all EEP samples was assessed by a SRB assay using CPE reduction [19, 20]. In brief, HeLa cells were seeded onto 96-well culture plates at a density of 3 × 10^4^ cells per well for 1 day. The culture medium was then removed and the plates were washed with PBS. Subsequently, 90 μL of diluted virus suspension containing CCID_50_ of the virus stock was put into the wells, and then 10 μL of MEM supplemented with 30 mM MgCl_2_ containing four to five concentrations (0.1–200 μg/mL) of each EEP sample in 0.1 % DMSO was added to produce an appropriate CPE within 2 days after infection. After incubation at 37 °C and 5 % CO_2_ for 2 days, the plates were washed once with 200 μL PBS. The 100 μL of 0.057 % (w/v) SRB in 1 % acetic acid solution was added to each well and left at room temperature for 30 min. The absorbance was recorded using a VersaMax microplate reader at 562 nm and at 620 nm reference. Ribavirin served as a positive control and negative controls consisted of the DMSO solution. Viral inhibition rate (VIR) (%) was calculated according to the formula [20]: % VIR = (*OD*_tV_ – *OD*_cV_)/(*OD*_cd_ – *OD*_cV_) × 100, where *OD*_tV_ is the optical density measured with a given concentration of the EEP sample in HRV infected cells; *OD*_cV_ is the optical density measured for the control untreated HRV infected cells; *OD*_cd_ is the optical density measured for the control untreated HRV uninfected cells.

### Intestinal bacterial strains and growth inhibitory assay

Five harmful bacteria, two nonpathogenic bacteria, six lactic acid-producing bacteria, and an acidulating bacterium used in this study are listed in Table 2. Stock cultures of the bacterial strains were stored on BHI broth (pH 7.6) containing 25 % glycerol (v/v) at −70 °C. The cultures of *Escherichia coli* ATCC 11775 and *Staphylococcus aureus* ATCC 12600 were incubated at 37 °C for 1 day under aerobic condition, while the cultures of the other 12 bacterial strains were incubated at 37 °C for 1 day in an atmosphere of 5 % H_2_, 15 % CO_2_, and 80 % N_2_ in a FA-6 anaerorator (serial no. 98072851) (Hirayama, Tokyo, Japan) [21]. For bioassay, bacterial suspensions containing 1 × 10^5^ colony-forming unit (CFU)/mL were prepared in EG agar using 24 h subcultures in BHI broth.

**Table 2:** List of human intestinal bacteria tested for growth inhibitory activity

Harmful or nonpathogenic bacteria	Beneficial bacteria
Gram-positive	Gram-positive lactic acid-producing bacteria
* Clostridium difficile* ATCC 9689	*Bifidobacterium bifidum* ATCC 29521
*Clostridium paraputrificum* ATCC 25780	*Bifidobacterium breve* ATCC 15700
*Clostridium perfringens* ATCC 13124	*Bifidobacterium infantis* ATCC 25962
*Staphylococcus aureus* ATCC 12600	*Bifidobacterium longum* ATCC 15707
	*Lactobacillus acidophilus* ATCC 4356
	*Lactobacillus casei* ATCC 393
Gram-negative	Gram-positive acidulating bacterium
* Bacteroides fragilis* ATCC 25285^a^	*Clostridium butyricum* ATCC 25779
* Escherichia coli* ATCC 11775^a^	
*Salmonella typhimurium* ATCC 13311	

^a^Nonpathogenic bacteria

A microtiter plate-based bioassay in sterile 96-well plates was used to determine the minimal inhibitory concentrations (MICs) of all EEP samples toward the organisms, as described by Sarker et al. [22]. In brief, initial EEP samples were prepared in DMSO, and a twofold dilution series was then formulated in 50 μL BHI broth. Subsequently, 10 μL bacterial suspension of each strain was added. Ciprofloxacin served as a positive control and was similarly formulated. Negative controls consisted of the DMSO solution only. Treated and control plates were incubated under the same conditions as those used for bacterial cultures for 24 h. Then, 10 μL of resazurin solution (270 mg resazurin in 40 mL sterile distilled water) was added to each well.

### Data analysis

MIC (mg/mL) was defined as the lowest concentration of EEP that visually inhibited bacterial growth using resazurin indicator. The BACE-1 inhibitory, AChE inhibitory, and free radical scavenging activity was expressed as 50 % inhibition concentration (IC_50_, μg/mL) of the EEP that is required to cause 50 % BACE-1, AChE, and DPPH inhibition, respectively. The antiproliferative activity was expressed as 50 % inhibition concentration (IC_50_, μg/mL) of the EEP that is required to reduce the viability of cells to 50 % compared to the controls. The IC_50_ values of the EEP samples were calculated using Prism 5 software program (GraphPad Software, La Jolla, CA). The IC_50_ values were considered to be significantly different from one another when their 95 % confidence limits did not overlap. Results were expressed as mean ± standard error (SE) of triplicate samples of three independent experiments. The Bonferroni multiple-comparison method was used to test for significant differences in total polyphenol and flavonoid contents among EEP samples using SAS 9.13 program (SAS Institute, Cary, NC). Correlation coefficient (*r*) analysis of the biological activities of EEP samples was determined using their IC_50_ or MIC values and their polyphenol or flavonoid contents in 20 Korean EEP samples.

## Results

### Total polyphenol and flavonoid contents of propolis samples

The total polyphenol and flavonoid contents in 20 Korean EEP samples were compared with those of Australian, Brazilian, and Chinese EEP ones (Table 3). The total polyphenol contents (*F* = 92.79; df = 22, 46; *P* < 0.0001) and flavonoid contents (*F* = 68.66; df = 22, 46; *P* < 0.0001) in 23 EEP samples significantly differed. The total polyphenol contents in 20 Korean EEP samples ranged from 48.5 to 238.9 mg GAE/g EEP. EEP samples from P10, P13, P3, and P1 showed higher total polyphenol contents (238.9–219.5 mg GAE/g EEP) than those from other regions. The total polyphenol content of EEP from P20 was the lowest of any of the propolis examined. The total polyphenol content of EEP samples from Brazil, China, and Australia was between 127 and 142 mg GAE/g EEP. The total flavonoid contents in Korean EEP samples ranged from 20.8 to 49.8 mg QE/g EEP. EEPs from P9, P20, and P2 showed higher total flavonoid contents (49.8–40.5 mg QE/g EEP) than those from other regions. The total flavonoid content of EEP from P17 was the lowest of any of the propolis examined. The total flavonoid content of EEP samples from China, Australia, and Brazil was between 33 and 53 mg QE/g EEP.

**Table 3:** Total polyphenol and flavonoid contents in 20 Korean, Australian, Brazilian, and Chinese propolis ethanol extracts

Sample	Total polyphenol (mg GAE/g EEP)	Total flavonoid (mg QE /g EEP)
P1	219.5 ± 5.31^a–c^	29.7 ± 0.77^f–h^
P2	148.0 ± 4.32^f–i^	40.5 ± 0.77^b,c^
P3	229.0 ± 4.82^a,b^	23.5 ± 0.15^i,j^
P4	202.4 ± 4.11^b–d^	30.4 ± 0.36^e–g^
P5	196.5 ± 4.28^c–e^	26.3 ± 0.57^g–i^
P6	135.9 ± 5.70^hi^	23.6 ± 0.38^i,j^
P7	205.6 ± 5.48^b–d^	35.9 ± 0.57^c–e^
P8	151.9 ± 5.01^f–h^	26.5 ± 0.56^g–i^
P9	205.0 ± 5.98^b–d^	49.8 ± 0.79^a^
P10	238.9 ± 4.61^a^	36.5 ± 0.61^b–d^
P11	132.3 ± 2.78^h,i^	25.3 ± 1.27^g–j^
P12	125.1 ± 5.37^i^	28.7 ± 0.17^f–i^
P13	233.5 ± 4.39^a,b^	28.3 ± 2.13^f–i^
P14	181.7 ± 4.20^d,e^	27.6 ± 0.29^f–i^
P15	183.2 ± 5.75^d,e^	26.6 ± 0.07^g–i^
P16	168.7 ± 5.68^e–g^	37.8 ± 2.02^b–d^
P17	190.3 ± 5.45^c–e^	20.8 ± 0.17^j^
P18	171.6 ± 6.21^e,f^	26.6 ± 0.20^g–i^
P19	148.3 ± 5.77^f–i^	24.4 ± 2.01^h–j^
P20	48.5 ± 4.08^j^	42.2 ± 1.75^b^
Australian	142.4 ± 3.61^g–i^	38.0 ± 0.90^b–d^
Brazilian	126.8 ± 4.12^h,i^	53.0 ± 0.22^a^
Chinese	132.1 ± 3.28^h,i^	32.5 ± 0.53^d–f^

*GAE* gallic acid equivalent, *QE* quercetin equivalent, *EEP* ethanol extract from propolis

Means followed by the same letter in the column are not significantly different (*P* = 0.05, Bonferroni method)

### In vitro BACE-1 inhibitory activity

Because BACE-1 is a critical enzyme in the amyloid precursor protein (APP) amyloidgenic pathway that generates β-amyloid, the main component of amyloid plaque in the brain of AD [23], the BACE-1 inhibitory activity of all EEP samples was elucidated using a fluorescence resonance energy transfer enzyme assay (Table 4). As judged by IC_50_ values, the BACE-1 inhibitory activity of 20 native Korean EEP samples ranged from 25.7 to 291.9 μg/mL. EEP from P7 (IC_50_, 26 μg/mL) was the most active propolis, followed by EEP samples from P2, P5, P1, P6, P10, and P16 (36.3–64.6 μg/mL). IC_50_ of EEP samples from China, Australia, and Brazil was between 116.4 and 476.5 μg/mL.

**Table 4:** BACE-1 inhibitory activity of 20 Korean, Australian, Brazilian, and Chinese propolis ethanol extracts

Sample	IC_50_, μg/mL (95 % CL)	Slope ± SE	χ^2a^	*P*-value
P1	55.8 (47.7–63.9)	1.8 ± 0.15	2.67	0.996
P2	36.3 (25.2–52.3)	0.9 ± 0.10	7.32	0.961
P3	70.0 (55.9–87.5)	1.1 ± 0.10	5.07	0.983
P4	101.1 (84.7–120.6)	1.6 ± 0.29	5.74	0.981
P5	52.9 (37.3–75.0)	0.9 ± 0.12	8.74	0.931
P6	58.2 (48.8–69.5)	1.5 ± 0.15	3.98	0.991
P7	25.7 (21.8–30.4)	1.2 ± 0.07	3.56	0.989
P8	99.1 (91.4–107.5)	1.6 ± 0.12	2.55	0.997
P9	115.4 (99.7–133.6)	3.4 ± 1.50	2.77	0.997
P10	61.4 (49.1–76.8)	1.4 ± 0.15	6.90	0.974
P11	291.9 (278.0–306.6)	1.3 ± 0.03	1.41	0.999
P12	140.0 (122.0–160.6)	1.2 ± 0.08	3.56	0.992
P13	122.5 (110.9–135.4)	2.5 ± 0.42	3.36	0.995
P14	140.5 (125.5–157.2)	2.2 ± 0.24	3.65	0.994
P15	141.1 (127.8–155.8)	2.0 ± 0.18	3.27	0.995
P16	64.6 (52.1–80.2)	1.7 ± 0.26	4.87	0.987
P17	96.7 (91.4–102.2)	2.3 ± 0.39	2.40	0.997
P18	117.5 (108.7–127.1)	2.0 ± 0.21	2.91	0.996
P19	97.8 (92.3–103.6)	2.3 ± 0.41	2.57	0.997
P20	128.7 (112.6–147.2)	1.3 ± 0.09	3.99	0.997
Australian	127.5 (114.2–142.3)	1.7 ± 0.15	3.60	0.993
Brazilian	476.5 (458.5–495.2)	1.6 ± 0.05	1.30	0.999
Chinese	116.4 (105.4–128.6)	2.7 ± 0.66	3.23	0.995

^a^Pearson’s chi-square goodness-of-fit test

### Human acetylcholinesterase inhibitory activity

Because AChE is one of the major targets of AD [16], the inhibitory activity of all EEP samples was compared with that of three anticancer agents (Table 5). As judged by IC_50_ values, the anti-AChE activity of 20 native Korean EEP samples ranged from 15.6 to 327.3 μg/mL. EEP from P2 (IC_50_, 15.6 μg/mL) was the most active propolis, followed by P20 (26.7 μg/mL) and P16 (33.9 μg/mL). They were significantly less active than either huperzine A, donepezil hydrochloride, or tacrine (IC_50_, 0.2–1.2 μg/mL). IC_50_ of EEP samples from China, Australia, and Brazil was between 147.0 and 242.9 μg/mL.

**Table 5:** Human acetycholinesterase inhibitory activity of 20 Korean, Australian, Brazilian, and Chinese propolis ethanol extracts and three commercial anti-Alzheimer’s disease agents

Sample	IC_50_, μg/mL (95 % CL)	Slope ± SE	χ^2a^	*P*-value
P1	70.0 (56.9–86.1)	1.0 ± 0.12	7.33	0.9113
P2	15.6 (11.2–21.6)	0.6 ± 0.05	3.04	0.9321
P3	55.7 (44.7–69.4)	1.1 ± 0.14	7.71	0.9018
P4	47.9 (39.0–59.0)	1.1 ± 0.14	6.96	0.9128
P5	119.9 (98.2–146.4)	2.4 ± 0.48	12.2	0.9066
P6	89.8 (80.1–100.6)	2.2 ± 0.23	6.75	0.9685
P7	48.0 (40.8–56.55)	1.2 ± 0.12	5.88	0.9406
P8	79.3 (72.6–86.6)	4.8 ± 0.67	6.67	0.9760
P9	103.2 (91.8–116.1)	2.6 ± 0.33	7.56	0.9665
P10	104.2 (89.2–121.7)	2.0 ± 0.26	8.59	0.9459
P11	123.4 (115.1–132.2)	2.6 ± 0.20	4.49	0.9878
P12	61.6 (56.4–67.2)	2.5 ± 0.25	5.57	0.9724
P13	101.6 (84.8–121.7)	2.0 ± 0.32	10.29	0.9242
P14	102.1 (84.8–122.8)	1.8 ± 0.26	9.87	0.9245
P15	104.9 (85.6–128.6)	1.8 ± 0.30	11.04	0.9070
P16	33.9 (26.5–43.2)	1.1 ± 0.13	6.39	0.9012
P17	130.8 (122.5–139.6)	4.1 ± 0.60	5.58	0.9842
P18	124.8 (101.7–153.2)	1.6 ± 0.22	9.94	0.9094
P19	327.3 (297.7–359.7)	2.6 ± 0.26	6.04	0.9785
P20	26.7 (20.7–34.5)	0.9 ± 0.09	4.85	0.9213
Australian	242.9 (231.9–254.5)	3.2 ± 0.22	3.39	0.9940
Brazilian	147.0 (139.4–155.1)	2.9 ± 0.19	3.66	0.9930
Chinese	230.9 (208.7–255.5)	3.6 ± 0.62	7.88	0.9696
Tacrine	1.2 (0.9–1.6)	0.4 ± 0.02	3.87	0.9712
Huperzine A	0.2 (0.1–0.3)	0.4 ± 0.03	5.33	0.9246
DH	0.4 (0.3–0.6)	0.3 ± 0.02	4.78	0.9466

*DH* donepezil hydrochloride

^a^Pearson’s chi-square goodness-of-fit test

### DPPH free radical scavenging activity

The antioxidant activity of all EEP samples was compared with that of an antioxidant agent ascorbic acid using a DPPH radical scavenging assay (Table 6). Based on IC_50_ values, the radical scavenging activity of 20 Korean EEP samples ranged from 43.4 to 269.0 μg/mL. EEP samples from P1 was the most active propolis, followed by EEPs from P3, P5, and P10. The inhibitory activity of the EEP samples was 3.1, 3.8, 4.0, and 4.2 times less active than that of ascorbic acid (IC_50_, 14 μg/mL), respectively. IC_50_ of EEP samples from Australia, Brazil, and China was between 73.8 and 179.0 μg/mL.

**Table 6:** DPPH free radical scavenging activity of 20 Korean, Australian, Brazilian, and Chinese propolis ethanol extracts and a commercial antioxidant agent ascorbic acid

Sample	IC_50_, μg/mL (95 % CL)	Slope ± SE	χ^2a^	*P*-value
P1	43.4 (36.6–51.4)	1.7 ± 0.16	5.32	0.983
P2	115.3 (100.1–132.9)	1.3 ± 0.11	4.29	0.989
P3	52.7 (44.0–63.3)	1.6 ± 0.17	5.71	0.981
P4	80.2(69.4–92.7)	1.3 ± 0.10	4.28	0.989
P5	56.1 (47.6–66.1)	1.5 ± 0.14	5.10	0.985
P6	97.4 (79.9–118.7)	1.2 ± 0.13	5.76	0.980
P7	73.5 (62.7–86.2)	1.4 ± 0.13	4.87	0.986
P8	159.9 (139.7–183.1)	1.9 ± 0.18	4.44	0.990
P9	81.7 (69.8–95.6)	1.9 ± 0.31	5.76	0.983
P10	58.3 (47.1–72.1)	2.2 ± 0.32	6.73	0.976
P11	161.1 (141.8–183.3)	1.9 ± 0.16	4.20	0.991
P12	235.0 (219.6–251.5)	1.3 ± 0.04	1.96	0.998
P13	67.2 (56.0–80.6)	1.7 ± 0.24	6.03	0.979
P14	135.4 (117.1–156.3)	1.7 ± 0.18	4.80	0.988
P15	98.7 (87.1–111.8)	1.4 ± 0.11	3.89	0.991
P16	81.6 (71.8–92.8)	1.4 ± 0.11	4.00	0.991
P17	74.0 (63.7–86.0)	1.6 ± 0.16	4.89	0.987
P18	96.2 (85.0–109.0)	1.5 ± 0.13	4.05	0.991
P19	202.0 (175.0–233.1)	1.5 ± 0.11	4.39	0.989
P20	269.0 (248.3–291.4)	1.5 ± 0.06	2.44	0.996
Australian	73.8 (49.3–110.5)	0.9 ± 0.12	9.76	0.922
Brazilian	148.1 (121.2–180.9)	1.6 ± 0.22	6.51	0.976
Chinese	179.0 (136.8–234.2)	1.8 ± 0.29	8.59	0.958
Ascorbic acid	14.0 (12.4–15.8)	1.8 ± 0.17	4.45	0.983

^a^Pearson’s chi-square goodness-of-fit test

### Antiproliferative effect on cancer cell lines

The antiproliferative activity of all EEP samples was compared with that of a commercial anticancer agent cisplatin using a MTT assay (Table 7). As judged by IC_50_ values, the antiproliferative activity of 20 Korean EEP samples toward PC-3 cell line ranged from 15.9 to 331.6 μg/mL. EEP from P2 was the most active propolis, followed by EEPs from P7 and P12. The inhibitory activity of the EEP samples was 3.8, 2.0, and 1.6 times more active than that of cisplatin (IC_50_, 61 μg/mL), respectively. IC_50_ of EEP samples from Australia, Brazil, and China was between 92.8 and 121.9 μg/mL. Toward MCF-7 cell line, IC_50_ of 20 Korean EEP samples was between 17.7 and 218.2 μg/mL. EEPs from P12 and P20 were the most active propolis and the antiproliferative activity of these samples did not differ significantly from that of cisplatin. IC_50_ of EEP samples from Australia, Brazil, and China was between 61.7 and 144.8 μg/mL. Toward A549 cell line, IC_50_ of 20 Korean EEP samples was between 6.5 and 365.3 μg/mL. EEPs from P1, P7, P8 and P9 (IC_50_, 6.46–11.0 μg/mL) were the most active propolis and the antiproliferative activity of these samples did not differ significantly from that of cisplatin. IC_50_ of EEP samples from Australia, Brazil, and China was between 152.2 and 452.8 μg/mL.

**Table 7:** Antiproliferative activity of 20 Korean, Australian, Brazilian, and Chinese propolis ethanol extracts and a commercial anticancer agent cisplatin toward three cancer cell lines

Sample						
	PC-3 cell		MCF-7 cell		A549 cell	
	IC_50_, μg/mL (95 % CL)	Slope ± SE	IC_50_, μg/mL (95 % CL)	Slope ± SE	IC_50_, μg/mL (95 % CL)	Slope ± SE
P1	54.0 (44.6–65.4)	0.7 ± 0.07	58.0 (50.6–66.4)	1.5 ± 0.14	6.5 (4.4–9.5)	0.8 ± 0.10
P2	15.9 (13.7–18.4)	1.2 ± 0.10	38.6 (33.6–44.3)	2.5 ± 0.35	20.2 (17.1–23.8)	0.7 ± 0.05
P3	49.8 (42.1–59.0)	0.9 ± 0.08	87.9 (71.0–108.8)	1.0 ± 0.13	164.5 (141.6–191.2)	0.7 ± 0.05
P4	69.7 (62.8–77.3)	0.9 ± 0.05	218.2 (184.9–257.4)	1.4 ± 0.14	61.1 (49.1–76.1)	0.9 ± 0.09
P5	44.2 (37.0–53.0)	1.1 ± 0.11	134.2 (106.1–169..6)	1.1 ± 0.14	40.9 (33.6–49.8)	0.9 ± 0.08
P6	58.2 (49.5–68.5)	1.1 ± 0.10	56.4 (49.7–64.0)	2.0 ± 0.22	16.3 (12.5-21.2)	0.9 ± 0.11
P7	29.9 (24.3–36.8)	1.2 ± 0.13	31.1 (28.2–34.4)	1.5 ± 0.09	9.8 (7.4–13.1)	0.7 ± 0.07
P8	59.6 (47.3–75.0)	1.1 ± 0.14	78.8 (73.6–84.4)	4.4 ± 0.42	11.0 (8.5–14.2)	0.7 ± 0.08
P9	77.9 (68.3–88.8)	0.9 ± 0.07	47.7 (41.8–54.4)	1.8 ± 0.19	9.9 (8.0–12.2)	1.0 ± 0.12
P10	228.8 (189.5–276.3)	0.7 ± 0.06	109.5 (90.2–132.9)	1.2 ± 0.15	365.3 (298.5–447.1)	1.2 ± 0.15
P11	44.0 (35.1–55.1)	1.4 ± 0.19	98.3 (82.6–116.9)	1.3 ± 0.15	65.3 (61.1-69.8)	4.6 ± 0.91
P12	36.7 (29.5–45.6)	1.1 ± 0.14	17.7 (15.5–20.2)	2.0 ± 0.23	70.7 (57.9–86.3)	0.5 ± 0.05
P13	106.6 (90.9–125.0)	0.7 ± 0.05	75.9 (68.3–84.5)	1.2 ± 0.08	262.1 (225.9–304.2)	1.2 ± 0.11
P14	75.3 (68.9–82.3)	1.4 ± 0.21	26.0 (22.8–29.6)	4.5 ± 1.17	176.1 (143.5–215.9)	1.2 ± 0.14
P15	70.2 (56.8–86.9)	0.7 ± 0.06	104.1 (91.3–118.7)	0.8 ± 0.05	73.5 (60.6-89.1)	5.9 ± 2.76
P16	331.5 (266.7–412.2)	0.6 ± 0.05	79.2 (70.8–88.6)	1.9 ± 0.017	127.1 (103.4–156.3)	1.0 ± 0.12
P17	331.6 (279.8–392.9)	0.8 ± 0.06	98.1 (81.3–118.3)	1.3 ± 0.015	86.7 (73.4–102.4)	2.2 ± 0.35
P18	76.4 (66.3–88.0)	1.0 ± 0.07	145.1(122.6–171.8)	1.2 ± 0.013	115.0 (97.6–135.6)	2.3 ± 0.37
P19	161.0 (131.4–197.1)	0.5 ± 0.03	49.8 (48.0–51.8)	3.9 ± 0.23	64.9 (54.1–78.0)	0.7 ± 0.07
P20	17.1 (13.9–21.1)	1.0 ± 0.11	19.8 (17.6–22.3)	2.2 ± 0.28	43.8 (38.8–49.3)	2.7 ± 0.39
AU	92.8 (78.5–109.7)	2.0 ± 0.29	144.8 (134.3–156.1)	3.2 ± 0.32	152.2 (126.3–183.3)	1.9 ± 0.30
BA	105.2 (96.8–114.3)	2.1 ± 0.16	61.7 (54.7–69.7)	1.8 ± 0.16	330.2 (287.7–379.1)	4.9 ± 0.89
CN	121.9 (115.1–129.1)	1.5 ± 0.06	122.5 (108.1–138.7)	1.7 ± 0.15	452.8 (361.8–566.5)	1.1 ± 0.13
CPN	61.5 (50.9–74.4)	1.1 ± 0.11	17.6 (15.3–20.3)	1.5 ± 0.15	6.2 (4.6–8.5)	1.0 ± 0.14

*AU* Australian; *BA* Brazilian, *CN* Chinese, *CPN* cisplatin

### Anti-human rhinovirus activity

The antiviral activity of all EEP samples was compared with that of the antiviral agent ribavirin using a SRB assay (Data not shown). EEP sample from Brazil was 5.9 and 5.1 times more toxic toward HRV-2 (IC_50_, 12.6 μg/mL) and HRV-4 (15.4 μg/mL) than ribavirin, respectively. IC_50_ of the other 22 EEP samples was >100 μg/mL toward two HRVs.

### Growth-inhibiting effect on intestinal bacteria

The growth inhibitory effects of all EEP samples on five harmful and two nonpathogenic intestinal bacteria as well as six lactic acid-producing bacteria and an acidulating bacterium examined were compared with those of the commercial antibiotic ciprofloxacin (Table 8). Responses varied according to bacterial species and propolis examined. As judged by MIC values, EEP samples from P6, P9, and P11 showed potent growth inhibitory activity toward *C. difficile* ATCC 9689, although the inhibitory activity of these compounds was less active than that of ciprofloxacin. The MIC of EEPs from P12, P9, and P19 was 1.84, 14.7, and 14.7 mg/mL toward *C. paraputiricum* ATCC 25780, *C. perfringens* ATCC 13124, and *S. enterica* serovar Typhimurium ATCC 13311, respectively (ciprofloxacin, 0.062, 0.031, and 0.125 mg/mL). Toward *B. fragilis* ATCC 25285, the MIC of EEPs from P8, P12, and P19 was 3.7, 1.84, and 1.84 mg/mL, respectively (ciprofloxacin, 0.062 mg/mL). The MIC of EEPs from P9, P12, P14, and P19 was 1.84 mg/mL toward *E. coli* ATCC 11775 (ciprofloxacin, 0.062 mg/mL). The MIC of EEPs from P8 and P19 was 14.7 mg/mL toward *B. bifidum* ATCC 29521 and *B. longum* ATCC 15707 (ciprofloxacin, 0.016 and 0.031 mg/mL). The MIC of EEPs from P12 and P19 was 14.7 mg/mL toward *B. infantis* ATCC 25962 and 14.7 and 7.4 mg/mL toward *C. butyricum* ATCC 25779, respectively (ciprofloxacin, 0.031 mg/mL). The other EEP samples were ineffective toward all bacterial strains examined (MIC, > 30 mg/mL).

**Table 8:** Minimal inhibitory concentrations (MICs) of Korean propolis ethanol extracts and a commercial antibiotic ciprofloxacin toward four harmful and two nonpathogenic intestinal bacteria

Test bacteria	No. active propolis	Propolis sample^a^ (MIC, mg/mL)
*C. difficile* ATCC 9689^b^	3	P6 (1.84), P9 (1.84), P11 (1.84), CF^d^ (0.031)
*C. paraputrificum* ATCC 25780^b^	1	P12 (1.84), CF^d^ (0.062)
*C. perfringens* ATCC 13124^b^	1	P9 (14.7), CF^d^ (0.031)
*S. aureus* ATCC 12600^b^	0	CF^d^ (0.031)
*B. fragilis* ATCC 25285^c^	3	P8 (3.7), P12 (1.84), P19 (1.84), CF^d^ (0.062)
*E. coli* ATCC 11775^c^	4	P9 (1.84), P12 (1.84), P14 (1.84), P19 (1.84), CF^d^ (0.062)
*S. enterica* Typhimurium ATCC 13311^c^	1	P19 (14.7), CF^d^ (0.125)
*B. bifidum* ATCC 29521^b^	1	P8 (14.7), CF^d^ (0.016)
*B. infantis* ATCC 25962^b^	2	P12 (14.7), P19 (14.7), CF^d^ (0.031)
*B. breve* ATCC 15700^b^	0	CF^d^ (0.031)
*B. longum* ATCC 15707^b^	1	P19 (14.7), CF^d^ (0.031)
*L. acidophilus* ATCC 4356^b^	0	CF^d^ (0.062)
*L. casei* ATCC 393^b^	0	CF^d^ (0.031)
*C. butyricum* ATCC 25779^b^	2	P12 (14.7), P19 (7.4), CF^d^ (0.031)

^a^The other Korean propolis samples and three foreign (Austrailian, Brazilian, and Chinese) samples were ineffective (MIC, > 30 mg/mL)

^b^Gram-positive bacteria

^c^Gram-negative bacteria

^d^Ciprofloxacin

## Discussion

Propolis contains a wide variety of phenolic compounds, mainly flavonoids, phenolic acids, and their esters [1, 2, 7]. A broad spectrum of biological properties of propolis [1, 3–5] is related to its phenolic composition in flavonoids and other phenolic compounds [1, 2, 5]. The flavonoid content of propolis is attributed to the different preferred regional plants collected by honey bees. Flavonoid chemistry and extensive biological activities, such as antioxidant, hepatoprotective, antimicrobial, anti-inflammatory and anticancer, have been well documented by Nijveldt et al. [24] and Kumar and Pandey [25]. The total polyphenol (TP) and flavonoid (TF) contents were reported in propolis from Algeria (TP, 55–279 mg/g, TF, 10–69 mg/g) [26], Argentina (TP, 257–393 mg/g, TF, 66–133 mg/g) [27], Brazil (TP, 94–149 mg/g, TF, 6–21 mg/g) [28], China (TP, 43–302 mg/g, TF, 8–162 mg/g) [6], Greece and Cyprus (TP, 80–338 mg/g; TF, 9–183 mg/g) [29], India (TP, 159.10 mg/g; TF, 57.25 mg/g) [30], Japan (TP, 53–431 mg/g; TF, 18–113 mg/g) [31], Morocco (TP, 0.74–91.22 mg/g; TF, 0.20–34.27 mg/g) [32], Poland (TP, 150–197 mg/g; TF, 36–62 mg/g) [33], Portugal (TP, 151–329 mg/g) [34], South Korea (TP, 85–283 mg/g; TF, 16–135 mg/g) [9], and Turkey (TP, 115–210 mg/g) [35]. In the current study, the total polyphenol and flavonoid contents of 20 native Korean EEP samples were between 49 and 232 mg/g and between 21 and 50 mg/g, respectively, although the polyphenol and flavonoid contents of propolis stated previously were 31 and 431 mg/g and between 3 and 183 mg/g, respectively. Certain Korean propolis samples examined possessed considerable total polyphenol and flavonoid contents, as compared with either Australian, Brazilian, or Chinese propolis samples examined. This finding indicates that propolis with high polyphenol and flavonoid contents have to be selected for commercial propolis products because of the biological significance of the polyphenols and flavonoids [1, 2, 5].

BACE-1 [36] and AChE [16] are two of the major targets of AD. There are two major neuropathological hallmarks of AD including neurofibrillary tangles consisting of hyperphosphorylated tau protein and extracellular amyloid plaques accumulation of β-amyloid peptides. β-Amyloid was generated via sequential proteolytic processing of APP by BACE-1 and γ-secretase in APP amyloidogenic processing pathway [37, 38]. BACE-1 is a prime drug target for inhibiting β-amyloid generation because it is responsible for initiating β-amyloid production [39]. No information, however, is available concerning the BACE-1 inhibitory activity of propolis and its constituents. Brazilian propolis improved cognitive function in the patients with mild cognitive impairment that was deemed as a prodromal stage of AD [40]. In addition, inhibition of AChE, responsible for the breakdown of acetylcholine in the neural synapse, is a possible strategy for treatment of AD, which is characterized by a decline in cognitive function and mental atrophy. Propolis with a high phenol content may be an alternative for prevention and/or retardation of AD symptoms because phenols and flavonoids inhibit AChE activity [41]. The anti-AChE activity was reported in propolis from India (IC_50_, 43.46 μg/mL) [42] and Morocco (43–743 μg/mL) [32]. In the current study, IC_50_ of 20 native Korean EEP samples was between 15.6–327.3 μg/mL and the anti-AChE activity of the EEPs was lower than that of either donepezil hydrochloride, tacrine, or huperzine A. Certain Korean propolis samples exhibited good BACE-1 inhibitory activity (IC_50_, < 100 μg/mL) and were more pronounced in the inhibitory activity than either Australian, Brazilian, or Chinese propolis examined. Many peptidomimetics and heterocyclic compounds have been evaluated as BACE-1 inhibitors [43, 44]. However, none of these have been successfully developed as anti-AD drugs. These results verify that the Korean propolis merit further study as a potential anti-AD agents.

Antioxidants, which scavenge free radicals such as superoxide (O_2_), hydroxyl (OH), and peroxyl (OOH, ROO) radicals, are known to possess an important role in preventing these free radical-induced diseases, such as aging, cardiovascular disease, rheumatoid arthritis, cancer, diabetes, and neurological disorders (AD and Parkinson’s disease) and inflammation [45]. The flavonoids are powerful antioxidants, capable of scavenging free radicals [24, 25]. The DPPH free radical scavenging activity was reported in propolis from Algeria (IC_50_, 19.4– > 50 μg/mL) [26], Argentina (25–37.5 μg/mL) [46], Brazil (17.13–83.60 μg/mL) [28], China (32 μg/mL) [47], Greece (138–1557 μg/mL) [48], India (70 μg/mL) [30], Morocco (8–1813 μg/mL) [32], and Portugal (6.22–52 μg/mL) [34]. In the current study, IC_50_ of 20 native Korean EEP samples were between 43 and 269 μg/mL, although IC_50_ of propolis stated previously was 6 and 1813 μg/mL. Certain Korean propolis samples exhibited good antioxidant activity (IC_50_, < 100 μg/mL) and the activity of these samples and Australian propolis sample did not differ significantly from each other. Our current finding indicates that the Korean propolis merit further study as a potential antioxidant, although the activity of these propolis samples was lower than that of ascorbic acid.

The antiproliferative activity of propolis and its constituents toward various cancer cell lines have been well documented by Watanabe et al. [12] and Chan et al. [49]. The antiproliferative activity toward PC-3 cell line was reported in propolis from Brazil (optimal dose, 40 μg/mL) [50] and India (IC_50_, 41.8–134.5 μg/mL) [51]. The antiproliferative activity toward MCF-7 cell line was reported in propolis from Cuba (1–25 μg/mL) [52], India (13 μg/mL) [42] and (26.88–104 μg/mL) [51], Indonesia (47.45 μg/mL) [53], Java (37.8–276.45 μg/mL) [54], Malta (67 μg/mL) [55], Portugal (36–182 μg/mL) [56], and Turkey (125 μg/mL) [57]. The antiproliferative activity toward A549 cell line was reported in propolis from Cuba (IC_50_, 35.48–99.5 μg/mL) [58], India (10 μg/mL) [42], and Tunisia (200 μg/mL) [59]. In the current study, IC_50_ of 20 native Korean EEP samples toward PC-3, MCF-7, and A549 cell lines were between 15.9 and 331.6 μg/mL, between 17.7 and 218.2 μg/mL, and between 6.5 and 365.3 μg/mL, respectively, although IC_50_ of propolis stated previously was between 41.8–134.5 μg/mL, between 1 and 276.45 μg/mL, and between 10 and 200 μg/mL. Certain Korean propolis samples exhibited good antiproliferative activity and were more pronounced in the activity than either Australian, Brazilian, or Chinese propolis samples. Of them, the activity of some propolis samples toward PC-3, MCF-7, and A549 cells was comparable to that of the anticancer agent cisplatin. Our current finding indicates that the Korean propolis merit further study as a potential anticancer agent.

The antimicrobial activity of propolis toward various pathogens have been well noted [3–5]. In humans, many species of bacteria (~500–1000 species) reside in the intestinal tract as a complex and dynamic ecosystem [60]. Major functions of the microbiota include metabolic activities that result in salvage of energy and absorbable nutrients, trophic effects on intestinal epithelia (cell proliferation and differentiation) and on immune structure and function, and protection of the colonized host against invasion by alien microbes (barrier effect) [61]. The microbiota might also be an essential factor in certain pathological disorders, including multisystem organ failure, colon cancer, and inflammatory bowel diseases [61]. In addition, prolonged treatment with antibiotics has often produced resistance to the drugs by pathogenic microorganisms [62], which is a major global public health problem in both developed and developing countries. Sometimes, serious side effects of antibiotics occur, such as taste disturbances, nausea, diarrhea, dyspepsia, headache, and angioedema, as well as disturbance of human gastrointestinal microflora [62, 63]. Alternative antibacterial agents with novel modes of action and low toxicity are urgently needed. It has been reported that propolis samples from Argentina [64], France [65], and Greece and Cyprus [29] is effective toward Gram-positive bacteria, exerting a limited activity toward Gram-negative bacteria. Boyanova et al. [66] reported that Bulgarian EEP inhibited 97 % (29 of 30 strains) of the nonspore-forming Gram-positive bacteria and 91 % (40 of 44 strains) of the Gram-negative bacteria.

In the current study, the Gram-positive and Gram-negative bacteria were observed to have different degrees of antimicrobial susceptibility to the EEP samples examined, although ciprofloxacin susceptibility among the bacterial groups did not differ greatly. EEP samples from P6, P9 and P11 and P12 exhibited pronounced growth inhibitory activity toward pathogenic Gram-positive bacteria *C. difficile* ATCC 9689 and *C. paraputiricum* ATCC 25780, respectively. EEP samples from P8 and P9 and P14 were active toward nonpathogenic Gram-negative bacteria *B. fragilis* ATCC 25285 and *E. coli* ATCC 11775, respectively, whereas EEP samples from P12 and P19 were active toward both Gram-negative bacteria. However, the propolis samples from Austrailia, Brazil, and China were ineffective toward five harmful bacteria and two nonpathogenic bacteria. Boyanova et al. [66] reported that Bulgarian EEP inhibited 35 % (7 of 20 strains) of *Clostridium* strains, including *C. difficile* (13 strains) and *C. perfringens* (2 strains), and 82 % (9 of 11 strains) of *B. fragilis* group strains. Ugur et al. [67] found growth inhibitory activity of EEP samples from Brazil and Bulgaria toward *E. coli* MU 8, MU 11, and MU 23, whereas no inhibitory activity of EEP samples from Argentina toward *E. coli* ATCC 25922 [64]. Interestingly, six lactic acid-producing bacteria were less susceptible than either harmful or nonpathogenic bacteria to Korean EEP samples. Detailed tests are needed to fully understand the different susceptibility of the EEPs to bacteria. This finding indicates that low concentrations of active Korean propolis extracts could be used in fermented or nonfermented products and drink products, aiming to selectively inhibit the growth of pathogenic bacteria.

Correlation between biological activity and phenolic compound contents has been well studied. High correlation between the total phenolic and flavonoids content and the free radical scavenging activity was reported in propolis samples from Argentina [46], Greece and Cyprus [29], Japan [31], and Poland [33]. Negative or no direct correlation between them was reported in propolis from Morocco [32] and in propolis from Brazil [68] and Greece [48], respectively. For the AChE inhibition, negative correlation between the total phenolic and flavonoids content and IC_50_ was reported in propolis samples from Morocco [32]. For the antiproliferative activity toward MCF-7 cell line, negative correlation between the total polyphenol content and IC_50_ was reported, whereas positive correlation between the total flavonoid content and IC_50_ was reported [56]. For the antimicrobial activity, strong correlation between the total phenolic and flavonoids content and MIC was reported in propolis from Spain [69], whereas no direct correlation between them was also reported in propolis from Brazil [68] and Greece and Cyprus [29]. In the current study, correlation coefficient (*r*) analysis showed that total polyphenol contents may be negatively correlated with DPPH free radical scavenging activity (*r* = −0.872) and total flavonoid content has no correlation with the activity (*r* = 0.071) (Table 9). No direct correlation between BACE-1 inhibition, AChE inhibition, or antiproliferative activity and total polyphenol or total flavonoid content in Korean EEP samples was found.

**Table 9:** Pearson correlation coefficients for total polyphenol or flavonoid content and biological activities

Activity	Correlation coefficient (*r*)	
	Total polyphenol	Total flavonoid
BACE-1 inhibition	−0.337	−0.194
Acetylcholinesterase inhibition	0.056	−0.413
DPPH free radical scavenging activity	−0.872	0.071
Antiproliferative activity		
PC-3	0.263	−0.076
MCF-7	0.386	−0.301
A549	0.453	−0.078

## Conclusions

Korean propolis-derived preparations could be useful in food and beverages to prevent various diseases in which free radicals, neurodegenerative causes, or pathogenic bacteria are implicated as propolis administration to humans does not lead to side effects [12]. For the practical use of the preparations as novel propolis-derived products to proceed, further research is needed to establish safety to humans and whether the biological activities are exerted in vivo after consumption of the products by humans. Propolis, administered orally to mice at levels up to 4000 mg/kg/day for 2 weeks, had no effect, although it has been identified clinically as an allergen [3]. Lastly, detailed tests are needed to understand how to improve biological potency and stability for eventual commercial development.
